# Gut Microbiota Dysbiosis in Functional Dyspepsia

**DOI:** 10.3390/microorganisms8050691

**Published:** 2020-05-08

**Authors:** Georgios Tziatzios, Paraskevas Gkolfakis, Ioannis S. Papanikolaou, Ruchi Mathur, Mark Pimentel, Evangelos J. Giamarellos-Bourboulis, Konstantinos Triantafyllou

**Affiliations:** 1 Hepatogastroenterology Unit, Second Department of Internal Medicine—Propaedeutic, Research Institute and Diabetes Center, Medical School, National and Kapodistrian University of Athens, “Attikon” University General Hospital, 124 62 Athens, Greece; g_tziatzios@yahoo.gr (G.T.); ispapn@hotmail.com (I.S.P.); 2 Department of Gastroenterology Hepatopancreatology and Digestive Oncology, Erasme University Hospital, Université Libre de Bruxelles, 1070 Brussels, Belgium; pgkolfakis@med.uoa.gr; 3 Medically Associated Science and Technology (MAST) Program, Cedars-Sinai, Los Angeles, CA 90048, USA; ruchi.mathur@cshs.org (R.M.); mark.pimentel@cshs.org (M.P.); 4 4th Department of Internal Medicine, Medical School, National and Kapodistrian University of Athens, 124 62 Athens, Greece; egiamarel@med.uoa.gr

**Keywords:** microbiota, dysbiosis, functional, dyspepsia

## Abstract

Functional dyspepsia (FD) is one of the most prevalent chronic functional gastrointestinal disorders. Several distinct pathophysiological mechanisms, including gastro duodenal motor disorders, visceral hypersensitivity, brain-gut interactions, duodenal subtle inflammation, and genetic susceptibility, have been implicated in the pathogenesis of the disease, so far. However, emerging evidence suggests that both quantitative and qualitative disturbances of the gastrointestinal microbiota may also be implicated. In this context, several studies have demonstrated differences of the commensal bacterial community between patients with FD and healthy controls, while others have shown that intestinal dysbiosis might associate with disease’s symptoms severity. Elucidating these complex interactions constituting the microbiota and host crosstalk, may eventually lead to the discovery of novel, targeted therapeutic approaches that may be efficacious in treating the multiple aspects of the disorder. In this review, we summarize the data of the latest research with focus on the association between gut microbiota alterations and host regarding the pathogenesis of FD.

## 1. Introduction

Functional dyspepsia (FD) is a frequent gastrointestinal (GI) disorder, characterized by epigastric pain or burning, postprandial fullness, or early satiation in the absence of structural disease on standard clinical and laboratory investigation, including upper GI endoscopy. Rome IV criteria identify three disease subtypes according to the predominant symptom pattern: postprandial distress syndrome (PDS), epigastric pain syndrome (EPS), and PDS-EPS overlap group, with variable prevalence worldwide [1]. *Helicobacter pylori (H. pylori)*-associated dyspepsia is now recognized as a distinct entity. The Kyoto global consensus report advocates that *H. pylori*-positive FD with a sustained response (>6–12 months) to eradication therapy is referred as *H. pylori*-associated dyspepsia and not FD [2]. Although a subset of FD patients (10–16%) may find symptomatic benefit after successful eradication therapy, only a minority will eventually remain asymptomatic on the long term, suggesting that *H. pylori* was not the primary cause of the dyspeptic symptoms [3,4]. Despite the latest advancements in the field, the disease’s aetiology and pathophysiology remain elusive and most probably multifactorial. Gastric sensorimotor abnormalities, brain–gut axis deregulation, visceral hypersensitivity, immune activation, altered epithelial barrier permeability, psychological stress, genetic background, and post-infectious low-grade duodenal inflammation are listed among the complex interactions thought to give rise to FD cardinal symptoms [4,5,6]. Aproximately 100 trillion commensal microorganisms residue synergistically in the human gut, including bacteria, archaea, fungi, eukaryotes and viruses [7]. The largest population is that of bacteria with more than 100 different species, further classified into four major phyla: Gram-positive *Firmicutes* producing short-chain fatty acids, Gram-negative *Bacteroidetes* producing hydrogen, as well as *Proteobacteria* and *Actionobacteria* [8]. This abundant and diverse microbial ecosystem represents a key element in maintaining the homeostasis of the host, since it acts as an effective and highly specialized barrier against pathogens, interacts with the immune system and contributes to the fermentative process of dietary and endogenous substrates [9]. Accumulating evidence have highlighted the potential role of gut microbiota dysbiosis—defined as any qualitative or quantitative alteration in their composition—in the pathogenesis of gastrointestinal and extra-gastrointestinal diseases, as well [10,11]. Dysbiosis has been consistently shown to associate with the onset and progression of symptoms in patients with irritable bowel syndrome (IBS), the other principal functional gastrointestinal disorder [12]. IBS frequently develops after an episode of infectious gastroenteritis or antibiotics intake, with evidence supporting the notion that gut microbiota composition significantly varies between IBS individuals and healthy ones [12]. Similarly to IBS, intestinal dysbiosis is an evolving concept dictating its further evaluation in patients with FD [5,13]. Given the fact that our treatment strategy for FD remains suboptimal, a detailed understanding of the mechanisms that may relate to the development of the disorder is pivotal in the search for novel therapeutic approaches [14]. The aim of this review was to present the latest literature data concerning the potential role of gut microbiota–host crosstalk in the pathogenesis of FD.

## 2. Methods and Methods

A search in PubMed database for studies published up to March 2020 in the English language was conducted using the following key words: (“gastrointestinal microbiome”[MeSH Terms] OR (“gastrointestinal”[All Fields] AND “microbiome”[All Fields]) OR “gastrointestinal microbiome”[All Fields] OR (“gut”[All Fields] AND “microbiota”[All Fields]) OR “gut microbiota”[All Fields]) AND (functional[All Fields] AND (“dyspepsia”[MeSH Terms] OR “dyspepsia”[All Fields])).

## 3. Role of Microbiota in FD Pathogenesis—Putative Pathophysiologic Mechanisms

**Figure 1:**
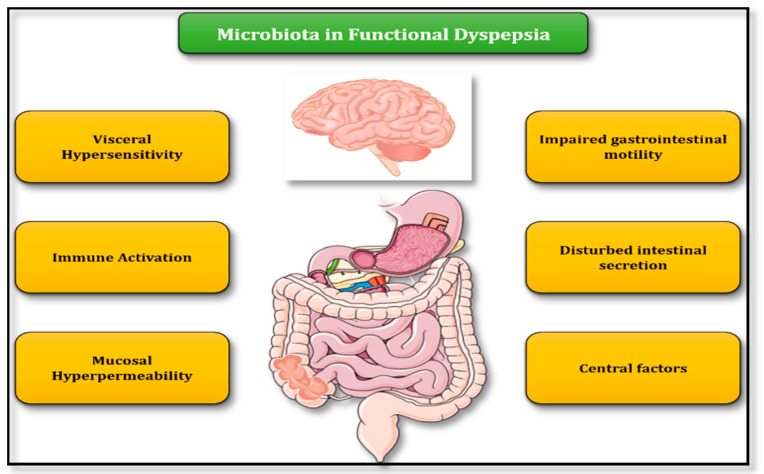
Putative mechanisms of gut microbiota involvement in FD pathogenesis.

Evidence from animal and clinical studies imply an intriguing role for intestinal flora in FD, through a number of pathogenic mechanisms which include impaired gastrointestinal motility, visceral hypersensitivity, immune activation, increased mucosal permeability, and central nervous system disorders [10] (Figure 1).

### 3.1. Abnormal Gastrointestinal Motility

Altered gastric sensorimotor function is thought to contribute to the pathophysiology of both FD and IBS [10]. Although evident, motility alterations (delayed gastric emptying, impaired gastric accommodation, hypersensitivity to distention), have been found to correlate poorly or not at all with FD symptoms [15]. Gut microbiota and gastrointestinal motility seem to be inextricably linked one to each other. On the one hand, intestinal motility disturbances can affect the number and composition of microbial commensal flora by establishing conducive intraluminal circumstances [16], while on the other hand the microbiota itself may pose certain impact on upper intestinal transit [17,18]. The latter can occur as a result of the prokinetic properties of various fermentative microbial products or metabolites. Among them, short chain fatty acids (SCFAs)—produced by dietary starches and carbohydrates fermentation mediated by gut bacteria—and bile acids (deconjugation and dehydroxylation of bile acids is regulated by gut bacteria) are those most well-studied, so far. More precisely, SCFAs produced by bacteria not only modulate duodenal bicarbonate secretion in FD, but at the same time their fast duodenal absorption may also influence luminal bacterial colonization suppression [19]. In addition, bacterial lipopolysaccharide produced by *Escherichia coli* (E. coli) has been found to induce a significant delay of gastric emptying [20], while *Bifidobacterium*—when used as probiotic—considerably enhances small bowel motility [21].

### 3.2. Intestinal Barrier Integrity

FD patients appear to have increased intestinal permeability of the duodenal mucosa [22]. This is thought to allow intraluminal triggers to initiate a both local and systemic immune cascade, which leads to altered neuronal signaling, generating the dyspeptic symptoms [23]. Commensal flora have a key twofold role: first, inducing intestinal barrier function maturation by promoting expression of multiple epithelial tight junction proteins (i.e., claudin-3). This was shown in an experimental study where an impaired intestinal barrier (lack of toll-like receptor adaptor protein MyD88 and claudin 3) effectively resumed its normal function after enteral administration of probiotics promoting claudin 3 expression [24]. Similarly, probiotics were also able to mediate the up-regulation of gene and protein expression of another significant element of tight junctions, namely zonula occludens 1 (ZO-1) [25]. Recent research also features the key role of zonulin—a precursor of haptoglobin-2—in maintaining the homeostasis of the intestinal mucosa. Zonulin is involved in regulation both of tight and cell–cell junctions responsible of the influx of dietary and microbial antigens via paracellular route of intestinal absorption. These data implicate zonulin as a master regulator of intestinal permeability, linked to the development of chronic inflammatory disorders [26]. Second, bacteria can even up-regulate the expression of genes involved in intestinal wall protein production; administration of probiotics resulted in altered expression levels of tight junction-related genes, including those producing occludin [27]. Research also indicates that their metabolites can also directly—without interfering neural pathways—mediate epithelium permeability, compromising intestinal barrier function [28]. It should be underlined that the abovementioned conclusions mainly derive from studies investigating the effects of probiotics on epithelial permeability; thus, may not reflect the normal situation or effect of commensals. Given the absence of studies, their results should be seen cautiously when interpreting these findings in the context of FD.

### 3.3. Immune System Activation and Low-Level Inflammation

Immune activation seen as low-grade mucosal inflammation, with duodenal mast cells and eosinophils infiltration has been described in FD [5,29]. Evidence suggest that the fundamental pathophysiologic event is a disturbance of gut-homing T lymphocytes population within a T-helper type 2 (Th2) or a Th17 immune response to luminal antigens, that eventually leads to disruption of the delicate mucosal homeostasis with dyspeptic symptom appearance [29]. Gut microbiota and their metabolites interfere with signaling through Toll-like Receptors (TLRs—TLR2, TLR4) and precipitate proinflammatory cytokines production and immune reaction [30]. Furthermore, their metabolites could also share inflammatory properties or exert direct effect on T-cell differentiation [31]. An alternative innovative disease model might be proposed for the subset of patients developing Post-infectious FD. Commonplace secondary to a bacterial gastroenteritis induced by ordinary pathogens, i.e., *Campylobacter jejuni, Salmonella, E.coli, Shigella* is the production of the cytolethal distending toxin (CdtB) [12]. Antibodies produced by the host against CdtB can cross-react—potentially through a molecular mimicry mechanism—with vinculin, a cytoplasmic cytoskeletal protein found in myenteric ganglia and interstitial cells of Cajal (ICC), playing a crucial part in gastrointestinal tract motility and contractility [32]. This allowed anti-CdtB and anti-vinculin circulating antibodies levels to be used as biomarkers to differentiate IBS-Diarrhea predominant type from inflammatory bowel disease (IBD) in different settings, with promising results [33]. Interestingly, higher serum titles of anti-CdtB were also observed in FD patients compared to healthy controls in a recent large Australian cohort study, indicating that a similar disease pathway might also apply for FD [34].

### 3.4. Disturbances in Intestinal Secretion

Intestinal secretion is partially regulated by molecules and metabolites (SCFAs and bile salts) produced by the bacteria flora [35]. In detail, bile acids may increase chloride secretion, while SCFAs participate in mucus, water and duodenal bicarbonate secretion [19,36]. Furthermore, serotonin, apart from being responsible for normal GI transit, has been also shown to contribute to visceral hypersensitivity by affecting intestinal permeability and has been associated to methane producing IBS subjects [37]. Gut microbiota may control serotonin secretion, thus featuring an alternative putative mechanism that might also implicated in FD pathogenesis [38].

### 3.5. Visceral Hypersensitivity

Although some of FD most burdensome symptoms i.e., pain, bloating, abdominal distention have been associated to excessive visceral hypersensitivity secondary to mechanical and chemical stimuli, evidence supporting the role of the bacterial community in this altered perception and sensation remain scarce [4,39]. Some data from experimental animal models suggest that gut microbiota might be implicated in the activity of central or peripheral neuronal pathways, while they could also produce molecules (i.e., nitric oxide, γ-aminobutyric acid) affecting sensation [40]. However, further data are needed to unveil a potential causal relationship.

### 3.6. Central Nervous System Factors

The functional link between gut and central nervous system (CNS) has been identified as an essential component of the FD pathogenesis [1]. Its disorders not only refer to an abnormal central modulation (brain to gut—activation of the hypothalamus pituitary-adrenal (HPA) axis affecting gut permeability, motility and secretion), but also involve altered intestinal signaling (gut to brain), thus, highlighting its bidirectional nature [23]. Gut microbiota fluctuations seem to relate with impaired mucosal surface integrity (through effect on TLRs and/or tight junction proteins and other inflammatory cytokines profile) [41]. The commensal bacterial community could also impress GI tract motility, once again via effect on TLR signaling, mediated by the pacemaker activity of the interstitial cells of Cajal [42]. It should be noted that bacteria produce metabolites (SCFAs) and neurotransmitters (gamma aminobutyric acid—GABA) that may also encumber brain function. Intestinal dysbiosis also causes production and release into the bloodstream of a pro-inflammatory endotoxine, lipopolysaccharide (LPS). LPS is a potent factor influencing CNS function and also leads to production of various other inflammatory cytokines that also affect physiological CNS activity, modulating neuropeptides synthesis [43].

## 4. *Helicobacter pylori* (*H. pylori*) and Gut Microbiota

The discovery of *Helicobacter pylori* (*H. pylori*) revolutionized our concept about gastroduodenal pathologies and more importantly gastric cancer. Beyond its undisputed carcinogenic potential in gastric epithelium that allocate the pathogen as class I carcinogen, it has been also evident that a mutual interaction between *H. pylori* and the gastric microbiota community is present [44,45]. One of the first studies aiming to clarify the potential differences in gastric microbiota between *H. pylori*-infected and non-infected subjects, found no difference in gastric bacterial composition at phylotype level, regardless *H. pylori* status [46]. However, this finding was not confirmed in studies that followed, where an increase in *Proteobacteria* and decrease in *Actinobacteria*, *Bacteroidetes*, and *Firmicutes* among *H. pylori*-positive patients was reported [47]. Another study from Mongolia using 16S rRNA gene amplicon sequencing, found that *H. pylori* infected subjects had significantly lower bacterial richness as well as Shannon and Simpson indices compared to those non-infected, while *H. pylori*-negative gastritis was associated with greater enrichment of *Firmicutes*, *Fusobacteria*, *Bacteroidetes* and *Actinobacteria* at phylum level [48]. An explanation for these striking discrepancies in microbiota composition could be attributed to differences of the recruited populations, in terms of dietary habits and gastric cancer risk. In this direction, a recent study detected significant differences in gastric microbiota among three distinct populations from a Southeast Asia region (the isolated Orang Asli of Malaysia, Myanmar residents and modern Malaysians), likely associated with the level of each population’s modernization [49]. At length, bacterial species richness and gastric microbiota diversity was more increased among less modernized individual and could suppress *H. pylori* growth. On the contrary, gastric microbiota composition varied significantly among modern participants with disparate gastric diseases. Greater abundance of *Cutibacterium acnes* in patients with non-ulcerative dyspepsia compared to those with peptic-ulcer disease was reported, suggesting that except *H. pylori* this particular bacterium may also induce gastritis.

Perhaps even more interesting are the data supporting that *H. pylori* has the potential to alter host metabolism, while the commensal microbiota can in fact attenuate the pathogen’s detrimental effect [50]. In a similar manner, *H. pylori* eradication therapy is not only able to prevent or even reverse gastric cancer development by interrupting the notorious atrophic gastritis - intestinal metaplasia - cancer cascade, but has been also found to affect the host’s metabolism by successfully restoring growth, weight and height along with increased serum acylated ghrelin level [51,52]. Of note, the most frequently used first-line treatment in FD, i.e., proton-pump inhibitors (PPIs) seems to cause minimal gastric microbiota alterations [53].

## 5. Data from Studies Evaluating Microbiota Dysbiosis in FD

As stated above, luminal dysbiosis may be involved in the pathogenesis of FD. Although the amount of available data supporting this is not directly comparable to that of IBS, the topic is likely to grow in importance as microbiota and imminent treatment approaches become a focal point for future research. Table 1 summarizes data from studies investigating microbiota alterations in FD [54,55,56,57,58,59].

Correlation of dysbiosis and FD was initially investigated in a prospective cohort study, comparing basic physiological properties of the gastric fluid (GF) and assessing the microbiota profile in 44 patients with FD and 44 healthy participants [54]. Authors reported significantly increased GF volume in FD patients, a finding that suggests disturbance or delayed gastric emptying. Next, using 16S rRNA gene sequencing analysis, they showed that the overall composition of the bacterial flora is different between the two groups. Moreover, the abundance of genus *Prevotella* and of *Bifidobacterium/Clostridium* was higher in FD than in healthy controls, respectively. Subsequent treatment of the FD patients with the probiotic strain of *Lactobacillus gasseri* OLL2716, resulted in restoration of the microbial community composition, while the abundance of *Prevotella* inversely correlated with the severity of PDS symptoms. In another comprehensive study using the same methodology, Igarashi and colleagues compared GF microbiota synthesis between 24 patients with FD and 21 age-matched and gender-matched healthy controls [55]. They found that bacterial composition was completely different between the two groups, even at the phylum level. In detail, microbiota of the FD group was characterized by increased *Bacteroidetes* to *Proteobacteria* ratio and total absence of *Acidobacteria*, while in the control group the ratio *Bacteroidetes* to *Proteobacteria* was decreased and *Acidobacteria* were present. As previously, treatment with probiotic *Lactobacillus gasseri* OLL2716 shifted these changes in microbiota analysis of patients with FD towards to that observed in the control group.

However, these results should be seen cautiously given that microbiota populations in GF aspirations are susceptible to the effect of gastric acid, bile acids and pancreatic enzymes. More importantly, these iterations were not designed to reveal information concerning the potential region-dependent interaction of mucosa-associated microbiota (MAM) in FD [23]. To overcome these hardships, Zhong et al. [56] in a pioneer small study assessed duodenal mucosal microbiota—using a specifically designed encased biopsy forceps—nine patients with FD and nine matched for age, sex, and body mass index controls. *Streptococcus* was the predominant genus in both groups, while the relative abundance of *Prevotella*, *Veillonella* and *Actinomyces* was significantly decreased in the FD group. Moreover, they noticed a negative correlation between duodenal mucosal bacterial load and quality of life, while as bacterial load increased, diversity decreased.

Trying to provide a detailed characterization of the gastric MAM communities in dyspeptic patients, Sterbini et al. [57] evaluated microbial populations of 24 patients using 16S rRNA gene pyrosequencing. Among patients tested negative for concurrent *Helicobacter pylori* infection and not receiving proton pump inhibitors, the relative abundance of *Bacteroidetes* and *Prevotellaceae* was increased, while that of *Proteobacteria* was decreased. Further insights into this enigmatic interplay between microbes and GI motility are provided by a study evaluating gastric and duodenal MAM in 26 consecutive FD patients and 10 controls [58]. Authors observed a significant negative correlation between the relative abundance of the genus *Veillonella* in duodenal but not gastric mucosa with gastric emptying time (Spearman’s rho (r) = −0.59, *p* < 0.005). Correlation between FD and microbiota was also investigated in a Japanese study, where mucosa samples from five different upper gut sites (oral cavity, esophagus, gastric body, gastric antrum and duodenum)—using a brush during endoscopy—in 11 FD and seven healthy subjects were collected [59]. Although the MAM α-diversity did not differ between the two groups, β-diversity differed, with the phylum *Firmicutes* being increased in FD patients across all biopsied sites. At the genus level, *Streptococcus* was significantly increased in FD and its relative abundance also positively correlated with symptoms severity. Finally, at a species level the relative abundance of OTU 90 (the most prevalent sequence of *Streptococcus infantis*) was positively correlated with PDS and EPS scores.

To summarize, intestinal dysbiosis may be associated with symptom generation or exacerbation in a subset of patients with FD through several putative mechanisms. Acknowledging the fact that current data cannot support a direct causative process that leads to development of FD, they undoubtedly show the way for future research that will lead to better comprehension of the microbiota role in FD.

## 6. Modulating Microbiota as Potential Treatment for FD

### 6.1. Probiotics

Probiotics are “live microorganisms which, when administered in adequate amounts, confer a health benefit on the host” and prebiotics as “a non-digestible food ingredient that beneficially affects the host by selectively stimulating the growth and/or the activity of one or a limited number of bacteria in the colon” [60]. In this regard, they could represent an alternative beneficial therapy for FD, targeting duodenal dysbiosis. Indeed, data from several individual studies have verified this hypothesis. In the double-blind, parallel-group, placebo-controlled, randomized, controlled trial conducted by Ohtsu and colleagues, 116 (*Helicobacter pylori*—negative) FD individuals were randomized to receive for a 12-week period either a daily yoghurt containing *Lactobacillus gasseri* OLL2716 or placebo (fermented milk product without *L. gasseri*) [61]. Authors observed similar impressions—assessed by a questionnaire where participants rated the severity of FD and accompanying symptoms—regarding the overall effect on gastric symptoms between the two groups (*p* = 0.073), but significantly higher elimination rates for FD symptoms in the group assigned to probiotics (17.3% vs. 35.2% of placebo, *p* = 0.048). This finding was observed in PDS but not in EPS subjects. However, a recent meta-analysis incorporating data exclusively from five randomized controlled studies refutes the aforementioned results [62]. Although FD symptoms improved after overall treatment with probiotics or prebiotics vs. placebo (relative risk (RR): 1.15 (95% CI: 1.01—1.30)), use of probiotics alone was not associated with significant improvement in FD symptoms (RR: 1.13; 95% CI: 0.99—1.28). Moreover, the authors could not provide any valuable input regarding the strain or species that might be more advantageous over the others. Thus, incorporating these particular agents as therapeutic approaches seems promising but requires further confirmation.

### 6.2. Antibiotics

Antibiotics have also been extensively used in treatment of gastrointestinal diseases associated with intestinal dysbiosis (i.e., IBS) [12]. Rifaximin is a non-absorbable antibiotic with unique pharmacokinetic properties and favorable safety profile that has established itself as an efficacious option for treating diarrhea predominant IBS [63]. Although its exact mechanism of action is not entirely known, it is generally considered as multimodal, including potential anti-inflammatory actions and altering proximal small bowel microenvironment [64]. Rifaximin has been already tested in a randomized trial, where 86 consecutive FD patients were assigned to receive rifaximin 400 mg or placebo [65]. After eight weeks, significantly more patients who had received rifaximin experienced adequate relief of both global dyspeptic symptoms (GDS) (78% vs. 52%), while rifaximin provided more frequently adequate relief of belching and post-prandial fullness/bloating (PPF) at week 4. Furthermore, female sex was associated with a more favorable response to rifaximin (adequate relief of GDS at week 4: 76% vs. 42%, *p* = 0.006; week 8: 79% vs. 47%, *p* = 0.008). Undoubtedly, these results may be an additional argument for contribution of bacteria in FD, but more data are warranted before its efficacy in treating this disorder is known.

### 6.3. Fecal Microbiota Transplantation

Fecal microbiota transplantation (FMT) has been at the spotlight during the last years, as an innovative treatment modality that could alter the natural history and outcomes in gastrointestinal diseases where gut dysbiosis is foreseen. By restoring gut microbial community with the use of healthy microbiota, FMT is an established treatment for recurrent *Clostridium difficile* infection (CDI), but has been lately tested also in IBS [66]. Thinking outside the box, we speculate that FMT might be a promising future enrichment of our therapeutic armamentarium for dyspeptic patients.

## 7. What Lies in the Future?

Nowadays, a growing body of evidence delineates the crucial role of gut microbiota in several physiological processes of the host, but also their implication in pathogenesis of various diseases. In order to improve our understanding regarding these mechanisms, pioneer, well-designed studies combining many different techniques (functional genomic, metagenomics, metabolomics, metatranscriptomics and metaproteomics) should be pursued. They will eventually allow the transition from simple taxonomic associations regarding abundant phyla and genera to functional phenotypes and cause–effect studies. An issue that usually remains underrated is the fact that gut microbiota represent a never-ending evolving community, subject to constant change in the form of several everyday factors, i.e., diet, body mass index, and medication (antibiotics, proton pump inhibitors), able to exert certain effect on its composition. Hence, all these individual factors as well as the significant heterogeneity in terms of study populations enrolled, criteria used to define FD, and the technique used to evaluate host–microbiota interactions represent additional challenges that should be addressed. Finally, it is imperative that future studies investigating potential therapeutic interventions (i.e., probiotics) move on from empirically administered therapies to an individualized mechanism-based diagnostic and therapeutic management model.

## Figures and Tables

**Table 1:** Microbiota analysis studies in Functional Dyspepsia.

Ref.	Population	Population Synthesis (FD/Controls, n)	Technique for Microbiota Identification	Principal Findings
**Gastric fluid aspirate**				
Nakae et al. [54]	Adult	44/44	16S rRNA gene sequencing	Higher levels of *Prevotella* in FD, inverse correlation between *Prevotella* abundance and severity of PDS-FD
Igarashi et al. [55]	Adult	21/21	16S rRNA gene sequencing	Higher *Bacteroidetes* > *Proteobacteria* abundance, absence of *Acidobacteria* in FD; lower *Bacteroidetes* < *Proteobacteria* abundance, presence of *Acidobacteria* in controls
**Mucosa-associated microbiota (MAM)**				
Zhong et al. [56]	Adult	9/9	16S rRNA gene sequencing	*Streptococcus* was the predominant genus in both control and FD; inverse relationship between abundance of *Streptococcus* and *Prevotella*, *Veillonella* and *Actinomyces*; negative correlation between bacterial load and quality of life
Sterbini et al. [57]	Adult	24	16S rRNA gene pyrosequencing	Higher levels of *Proteobacteria, Firmicutes, Bacteroidetes, Fusobacteria,* and *Actinobacteria*; higher levels of *Firmicutes* (*Streptococcaceae*) and *Streptococcus* in treatment with proton pump inhibitors
Shanahan et al. [58]	Adult	26/10	16S rRNA gene sequencing	Negative correlation between abundance of *Veillonella* and gastric emptying time
Fukui et al. [59]	Adult	11/7	16S rRNA gene sequencing	Higher levels of *Firmicutes* in FD compared to healthy controls; at genus level, higher levels of *Streptococcus* in FD; *Streptococcus* relative abundance positively correlated with symptoms

FD: Functional Dyspepsia; C: Controls (as defined in each study).
